# Systems genetics of sensation seeking

**DOI:** 10.1111/gbb.12519

**Published:** 2018-10-08

**Authors:** Price E. Dickson, Tyler A. Roy, Kathryn A. McNaughton, Troy D. Wilcox, Padam Kumar, Elissa J. Chesler

**Affiliations:** ^1^ Center for Systems Neurogenetics of Addiction The Jackson Laboratory Bar Harbor Maine

**Keywords:** addiction, alcohol, BXD, *Gnb1*, *Mef2c*, mouse, operant sensation seeking

## Abstract

Sensation seeking is a multifaceted, heritable trait which predicts the development of substance use and abuse in humans; similar phenomena have been observed in rodents. Genetic correlations among sensation seeking and substance use indicate shared biological mechanisms, but the genes and networks underlying these relationships remain elusive. Here, we used a systems genetics approach in the BXD recombinant inbred mouse panel to identify shared genetic mechanisms underlying substance use and preference for sensory stimuli, an intermediate phenotype of sensation seeking. Using the operant sensation seeking (OSS) paradigm, we quantified preference for sensory stimuli in 120 male and 127 female mice from 62 BXD strains and the C57BL/6J and DBA/2J founder strains. We used relative preference for the active and inactive levers to dissociate preference for sensory stimuli from locomotion and exploration phenotypes. We identified genomic regions on chromosome 4 (155.236‐155.742 Mb) and chromosome 13 (72.969‐89.423 Mb) associated with distinct behavioral components of OSS. Using publicly available behavioral data and mRNA expression data from brain regions involved in reward processing, we identified (a) genes within these behavioral QTL exhibiting genome‐wide significant *cis*‐eQTL and (b) genetic correlations among OSS phenotypes, ethanol phenotypes and mRNA expression. From these analyses, we nominated positional candidates for behavioral QTL associated with distinct OSS phenotypes including *Gnb1* and *Mef2c*. Genetic covariation of *Gnb1* expression, preference for sensory stimuli and multiple ethanol phenotypes suggest that heritable variation in *Gnb1* expression in reward circuitry partially underlies the widely reported relationship between sensation seeking and substance use.

## 1 INTRODUCTION

Substance abuse is a heritable disease with devastating effects on individuals and society.^1^, ^2^ Variance in the initial choice to experiment with addictive substances and the progression to addiction that is observed in some users can be partially explained by the multifaceted sensation seeking personality trait.^3^ Similar phenomena have been observed in preclinical studies using rodents: mice and rats which exhibit high exploration in a novel environment or preference for a novel environment over a familiar one also exhibit potentiation of substance use or addiction‐like behavior.^4^, ^5^, ^6^, ^7^, ^8^ Genetic correlations among sensation seeking and substance use indicate shared biological mechanisms,^9^ but the genes and networks underlying these relationships remain elusive. One reason for this may be the challenges of measuring the phenotypically complex sensation seeking trait in rodents using conventional sensation seeking assays which depend on locomotor behavior. A complementary approach is to measure an intermediate sensation seeking phenotype that (a) directly indexes the fundamental psychological drive that is shared by sensation seeking and substance use, (b) allows dissociation of biological mechanisms affecting motoric behaviors from those affecting sensation seeking and drug use and (c) is directly comparable across species.^10^, ^11^, ^12^

In this regard, an intriguing hypothesis is that heritable variation in the homeostatic set point of sensory stimulation^13^ underlies the observed relationship between sensation seeking and substance use in humans, and that these fundamental biological mechanisms are conserved across species. This hypothesis is supported by human^14^, ^15^ and mouse^16^, ^17^ studies in which operant sensation seeking (OSS), an index of an individual's preferred level of sensory stimulation,^18^ covaries with addiction‐related behaviors and is driven by reward‐system circuitry. In the OSS paradigm, the human participant or rodent subject is placed into an environment devoid of sensory stimulation and is given the opportunity to perform an operant response that introduces visual stimulation, auditory stimulation, tactile stimulation or some combination of these stimuli into the environment. Requests for sensory stimulation in the OSS paradigm provide a precise index of an organism's preferred level of sensory stimulation. Moreover, use of an operant paradigm allows the dissociation of variation in preference for sensory stimulation from variation in motoric behaviors. Thus, natural variation in an organism's preferred level of sensory stimulation provides an intermediate phenotype of sensation seeking which can be measured similarly in humans, nonhuman primates and rodents. Preclinical identification of genes and polygenic networks underlying this trait may provide a more direct window onto the biological mechanisms shared by sensation seeking and substance use that are relevant across species.

Combined use of the OSS paradigm in mice^19^ and a systems genetics approach^20^ allows discovery of the genetic and genomic mechanisms underlying heritable variation in the preferred level of sensory stimulation in the absence of a priori hypotheses about these mechanisms. Recombinant inbred (RI) mouse populations,^21^ sets of isogenic strains derived from a cross of inbred founder strains, are particularly suitable for systems genetics studies because individual animals within an RI strain are genetically identical and, consequently, reproducible. Therefore, data from multiple experiments and laboratories can be integrated over time enabling discovery of novel genetic relationships among behavioral phenotypes, molecular phenotypes or their combination. The BXD strains^22^, ^23^ are the largest and most extensively characterized RI population, and much of these data are publicly available on the GeneNetwork database (www.genenetwork.org).^24^

In the present study, we quantified the acquisition and maintenance of OSS in male and female mice from 62 BXD strains and the C57BL/6J and DBA/2J founder strains. Using these data, we performed QTL mapping to identify regions of the genome associated with OSS phenotypes. We used relative preference for the active lever and inactive control lever to dissociate QTL associated with variation in the preference for sensory stimuli from those associated with unrelated phenotypes affecting lever pressing such as locomotor hyperactivity or motor deficits. We then used systems genetics techniques including eQTL mapping and genetic covariation of gene candidate mRNA expression, OSS phenotypes and substance use phenotypes to identify candidate genes driving OSS and to assess the possibility of pleiotropic effects of these gene candidates on both OSS and substance use phenotypes.

## 2 MATERIALS AND METHODS

### 2.1 Subjects

OSS was assessed in male and female mice from 62 BXD strains and the C57BL/6J (B6) and DBA/2J (D2) founder strains (N = 247; approximately 2 males and 2 females per strain). The following BXD strains were tested: BXD1/TyJ, BXD2/TyJ, BXD6/TyJ, BXD9/TyJ, BXD11/TyJ, BXD13/TyJ, BXD14/TyJ, BXD15/TyJ, BXD16/TyJ, BXD19/TyJ, BXD20/TyJ, BXD21/TyJ, BXD27/TyJ, BXD28/TyJ, BXD29/TyJ, BXD31/TyJ, BXD32/TyJ, BXD33/TyJ, BXD34/TyJ, BXD38/TyJ, BXD39/TyJ, BXD40/TyJ, BXD42/TyJ, BXD43/RwwJ, BXD44/RwwJ, BXD45/RwwJ, BXD48a/RwwJ, BXD49/RwwJ, BXD50/RwwJ, BXD51/RwwJ, BXD53/RwwJ, BXD55/RwwJ, BXD56/RwwJ, BXD60/RwwJ, BXD61/RwwJ, BXD62/RwwJ, BXD63/RwwJ, BXD64/RwwJ, BXD65/RwwJ, BXD65a/RwwJ, BXD65b/RwwJ, BXD66/RwwJ, BXD67/RwwJ, BXD68/RwwJ, BXD69/RwwJ, BXD70/RwwJ, BXD71/RwwJ, BXD73/RwwJ, BXD73a/RwwJ, BXD74/RwwJ, BXD75/RwwJ, BXD77/RwwJ, BXD79/RwwJ, BXD81/RwwJ, BXD83/RwwJ, BXD86/RwwJ, BXD87/RwwJ, BXD90/RwwJ, BXD98/RwwJ, BXD100/RwwJ, BXD101/RwwJ, BXD102/RwwJ.

We prioritized historical BXD strains (BXD1/TyJ‐BXD42/TyJ) because a large pool of publicly available behavioral and gene expression data exists for these strains. The expanded BXD strains (BXD43/RwwJ‐BXD102/RwwJ) were selected based on availability at The Jackson Laboratory (JAX; Bar Harbor, Maine) at the time. We limited the number of strains to 64 because this number enabled counterbalancing based on the available number of operant conditioning chambers (n = 16) and total number of groups (n = 4) that could be tested in a single day. Specifically, the use of 64 strains allowed for the testing of one mouse per strain per cohort. Moreover, 64 strains provided sufficient statistical power to identify significant behavioral QTL and genetic correlations.

Experimental mice were obtained from the mouse production facility at JAX at 6 weeks of age and transferred to the JAX housing and phenotyping facility. Mice were group housed in duplex polycarbonate cages (Thoren Caging Systems, Inc; Hazleton, Pennsylvania; Maxi‐Miser Duplex II Mouse Cage) prior to testing at which point they were individually housed. A Nestlet and Shepherd Shack were provided in each cage for enrichment. The lid of each cage was fitted with a filtered top which reduced cross‐cage odor exposure when mice were removed from the ventilated racks for testing. Mice were maintained in a climate‐controlled room under a standard 12:12 light‐dark cycle (lights on at 0600 hours). Bedding was changed weekly and mice were provided free access to food (NIH31 5K52 chow, LabDiet/PMI Nutrition, St. Louis, Missouri) and acidified water. All procedures and protocols were approved by the JAX Animal Care and Use Committee and were conducted in compliance with the National Institutes of Health Guidelines for the Care and Use of Laboratory Animals.

### 2.2 Apparatus

OSS data were collected using 16 Med Associates (St. Albans, Vermont) operant conditioning chambers (ENV‐307W) enclosed in sound attenuating cubicles (ENV‐022V). The floor of each chamber consisted of bars which were covered by a single piece of acrylic to facilitate cleaning and mouse ambulation. Two retractable response levers (ENV‐312‐2W) were mounted to the left and right sides of the front wall and were positioned 18 mm above the chamber floor and 28 mm away from adjacent walls. A stimulus light (ENV‐321W) was mounted above each lever. A pellet receptacle (ENV‐303W) was positioned on the front wall 3 mm above the chamber floor equidistant between the two levers. The pellet receptacle was connected to a pellet dispenser (ENV‐203‐20). Pellets were not dispensed during the OSS procedure. A house light (ENV‐315W) was mounted on the rear wall of the chamber. Operant conditioning chambers were controlled by a Med Associates control unit using MED‐PC IV software. The OSS program was written in‐house in MEDState notation.

### 2.3 Behavioral testing

Mice were tested in 72‐minute sessions at the same time daily 7 days per week. Each session began with the illumination of the house light and extension of the two response levers. For each mouse, the right or left lever was defined as the active lever and the opposite lever was defined as the inactive lever. Active lever side was counterbalanced across strain, sex and cohort. Mice were tested in cohorts of 64 with one mouse per strain tested in each cohort. Half of the mice in each cohort were males and half were females. Within each cohort, the 64 mice were randomly assigned to a testing group (1‐4) and, within each group, an operant conditioning chamber (1‐16).

Mice were tested for 19 sessions on a fixed‐ratio 1 operant schedule in which a single active lever press resulted in a combination auditory‐tactile‐visual reward. The auditory and tactile components of the reward were accomplished by retraction, followed by immediate extension, of both the active and inactive levers. Concomitant with lever retraction, the house light was extinguished and the stimulus lights above the active and inactive levers were rapidly illuminated and extinguished (ie, flashed) to provide the visual component of the reward. Flash duration (1, 2, 4 or 8 seconds) and frequency (5, 2.5, 1.25 or 0.625) were randomized independently across rewards. The house light was reilluminated once the flashing of the stimulus lights had terminated. Throughout the entire session, inactive lever presses were recorded but had no consequences. There was no maximum number of reinforcers that could be earned during sessions; sessions terminated only after the 72‐minute session time had elapsed.

Number of active lever presses, number of inactive lever presses and number of rewards were collected on each of the 19 sessions across 12 6‐minute blocks. During reward delivery, active and inactive lever presses were counted but did not deliver additional rewards. Therefore, number of active lever presses would increase without concomitant increase in number of rewards if those presses occurred during reward delivery. Active lever preference was calculated as percentage of active lever presses relative to total lever presses during the entire session and separately during each of the 12 blocks.

### 2.4 Systems genetics analysis and gene candidate prioritization

We performed statistical analysis of behavior, genetic correlations, QTL mapping and gene candidate prioritization using previously reported methods.^9^ We used GeneNetwork^24^ to perform whole genome interval mapping of OSS phenotypes. We used one‐way or factorial analysis of variance (anova) to assess effects of sex and allele (B6 and D2) at the peak of identified QTL on OSS phenotypes. When performing repeated measures anova, the assumption of homogeneity of variance across groups and sessions was assessed using Mauchly's test of sphericity. The Huynh‐Feldt correction was used when this assumption was violated. Fisher's Least Significant Difference procedure was used when performing multiple comparisons, and the criterion for statistical significance was *P* < 0.05. Effect size for anova was reported as partial eta squared (*η*_p_^2^).

We used Mouse Genome Informatics (www.informatics.jax.org)^25^ and GeneNetwork to identify and prioritize gene candidates located within the two logarithm of odds (2‐LOD) confidence interval (CI) of QTL. Positional candidates were those genes that (a) were located within the 2‐LOD CI of a behavioral QTL, (b) exhibited significant *cis*‐eQTL in brain regions involved in reward processing^26^ including the ventral tegmental area (VTA), nucleus accumbens (NAc), prefrontal cortex (PFC) or hippocampus (HIPP) (GeneNetwork accession IDs: GN228, GN156, GN135 and GN112) and (c) exhibited mRNA expression (in the same region as a significant *cis*‐eQTL) that covaried with the phenotype used to map the behavioral QTL. Positional candidates were further prioritized based on the strength of these relationships, on the number of regions in which significant *cis*‐eQTL and significant covariation of phenotype and mRNA expression were observed, and published studies involving perturbation of potential gene candidates.

## 3 RESULTS

### 3.1 Statistical analysis of OSS

To assess performance on the OSS assay, we performed repeated measures anovas using number of lever presses, number of rewards or active lever preference as dependent measures. Strain and sex were between‐subjects factors. Session (1‐19) or block (1‐12) was a within‐subjects factor. When number of lever presses was used as the dependent measure, lever (active or inactive) was used as a second within‐subjects factor. About half of the mice (50.61%; n = 125) failed to press both the active and inactive lever on at least one of the 19 sessions (number of sessions on which mice failed to respond: 1 to 18; *M* = 7.62, SD = 5.21). On the final testing session, 49 mice (19.8% of all tested mice) failed to press both the active and inactive lever. For the 125 mice that failed to press a lever on at least one session, we imputed active lever preference scores using the multiple imputation procedure with 50 imputations.^27^

#### 3.1.1 Lever presses and rewards

**Figure 1:**
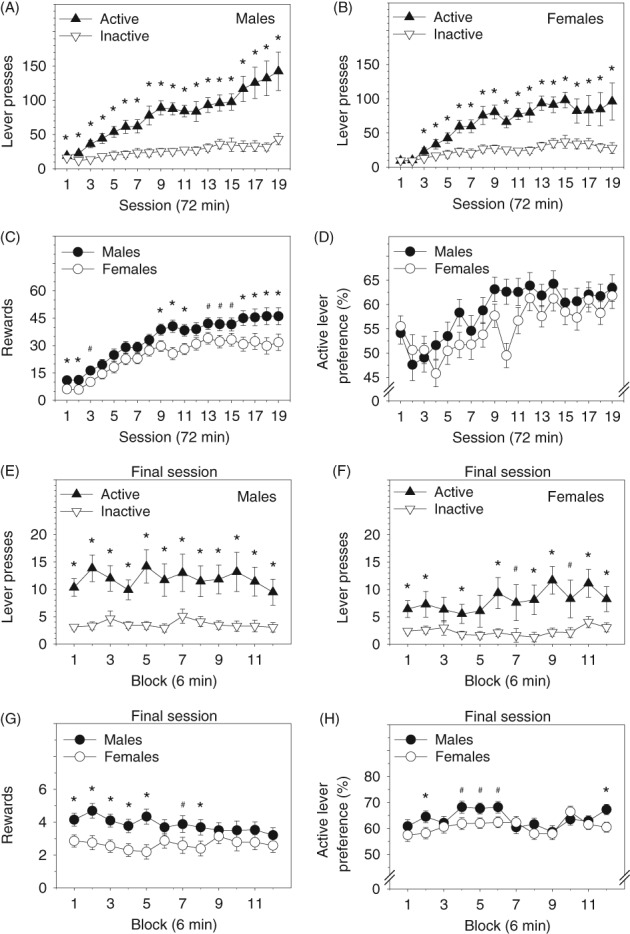
Operant sensation seeking in the BXD recombinant inbred mouse panel. A, B, Mice rapidly learned to press the active lever to receive a combination auditory‐tactile‐visual reward. Males learned to discriminate between the active and inactive levers more rapidly than females. C, Males received significantly more auditory‐tactile‐visual rewards than females during many of the testing sessions. D, Preference for the active lever increased significantly across sessions until it stabilized on sessions 11 to 19 in both males and females. E‐H, Response patterns on the final session indicate modest within‐session variation across blocks but do not indicate within‐session habituation of the reinforcing effectiveness of the auditory‐tactile‐visual stimuli. *P < 0.05; #*P* < 0.10

Mice rapidly learned to lever press for the combination auditory‐tactile‐visual reward (Figure 1A,B) as indicated by a significant session × lever interaction (*F* (4.76, 571.78) = 8.52, *P* = 1.53 × 10^−7^, *η*_p_^2^ = 0.066). Lever pressing was influenced by strain and sex as indicated by a significant strain × session interaction (*F* (310.09, 590.66) = 1.59, *P* = 7.19 × 10^−7^, *η*_p_^2^ = 0.46), strain × sex interaction (*F* (62, 120) = 1.79, *P* = 3.19 × 10^−3^, *η*_p_^2^ = 0.48) and strain × lever interaction (*F* (63, 120) = 2.53, *P* = 6.00 × 10^−6^, *η*_p_^2^ = 0.57). Males learned to lever press significantly more rapidly than females (Figure 1A,B) and received significantly more rewards on sessions 1 and 2 (Figure 1C). Males also received more rewards than females on many sessions during the second half of the OSS assay (Figure 1C). Number of active presses does not equal number of rewards because the active presses variable includes presses which resulted in a reward and presses that occurred during reward delivery (which did not result in a reward). Notably, active lever presses and rewards were almost perfectly genetically correlated (total active lever presses and rewards across 19 sessions: *rho* = 0.96, *P* = 1.00 × 10^−16^). Post hoc tests indicated that number of active lever presses and rewards were stable (ie, did not differ significantly) across the final four sessions in both males and females (*P* > 0.05 for all tests). In males, there was a trend toward increased active lever pressing during the final four sessions that approached but did not reach statistical significance (session 16 vs 19: *P* = 0.052).

To assess habituation of reinforcer effectiveness,^10^, ^11^ we examined within‐session lever pressing and reward delivery on the final testing session (Figure 1E‐G). The relationship of strain, block, sex and lever on lever pressing was complex as indicated by a significant strain × block × sex × lever interaction (*F* (682, 1320) = 1.14, *P* = 2.24 × 10^−2^, *η*_p_^2^ = 0.37). Number of active lever presses was significantly greater than number of inactive lever presses on all blocks in males (Figure 1E), and most blocks in females (Figure 1F). Males received significantly more rewards than females on early, but not later blocks (Figure 1G). In both males and females, post hoc tests indicated that number of lever presses did not decrease significantly across the 12 blocks (block 1 vs 12: *P* > 0.05) (Figure 1E,F).

#### 3.1.2 Active lever preference

Active lever preference increased significantly across the 19 OSS sessions as a function of strain as indicated by a significant strain × session interaction (*F* (1134, 2160) = 1.15, *P* = 2.55 × 10^−3^, *η*_p_^2^ = 0.37) (Figure 1D). The main effect of sex and interactions involving sex were not significant. Post hoc tests indicated that active lever preference was stable (ie, did not differ significantly) across the final nine sessions in both males and females (*P* > 0.05 for all tests).

To assess habituation of reinforcer effectiveness, we examined active lever preference within‐session on the final testing session (Figure 1H). The interaction of strain, block and sex was statistically significant (*F* (682, 1320) = 1.12, *P* = 3.54 × 10^−2^, *η*_p_^2^ = 0.36). Active lever preference was significantly greater in males than females on two blocks. Males, but not females, exhibited a modest but statistically significant increase in active lever preference across blocks (block 1 vs 12: *P* < 0.05).

### 3.2 QTL mapping of OSS phenotypes

We initially performed whole genome interval mapping for active lever presses, rewards and active lever preference on the final session of the OSS assay (ie, session 19). We chose to perform mapping on the final session because performance had stabilized for these variables by that point. Specifically, number of active lever presses and number of delivered rewards did not differ significantly across sessions 16 through 19 in males or females (Figure 1A‐C). Preference for the active lever did not differ significantly across sessions 11 through 19 in males or females on (Figure 1D). Note that preference for the active lever stabilized significantly earlier than active lever pressing and rewards variables. This was possible because the maximum value of percentage active lever preference is 100, whereas the value of active lever presses has no maximum value. Therefore, mice with a strong preference for the active lever could continue to increase active lever pressing over time with minimal increase in active lever preference.

Mapping lever pressing, reward and active lever preference variables on the final session showed a single genome‐wide significant behavioral QTL on chromosome 4 (*Oss1*) associated with active lever preference. Following identification of this QTL, we performed whole genome interval mapping for all variables on sessions 1 to 18 with the goal of identifying QTL associated with the process of acquiring the lever pressing response for the combination auditory‐tactile‐visual reward. On multiple sessions across the first 9 days of testing we identified the same genome‐wide significant behavioral QTL on chromosome 13 (*Oss2*) that was associated with lever pressing (ie, number of active lever presses, number of inactive lever presses and number of rewards) but not active lever preference. This QTL on chromosome 13, as well as the QTL on chromosome 4 associated with active lever preference, are described in Sections 3.2.1 and 3.2.2.

In a second analysis, we excluded all mice that did not acquire the lever pressing response for the auditory‐tactile‐visual reward (n = 136). Acquisition criteria were three consecutive sessions with ≥10 active lever presses and ≥70% active lever preference. In our previous studies^6^, ^9^, ^28^ we have used similar criteria to define acquisition of a lever pressing response for a cocaine reward. We calculated strain means from the OSS data after dropping these mice and attempted to map QTL. We did not identify genome‐wide significant QTL using this data set. This was likely due to the reduced statistical power from the combination of reduced sample size and reduced between‐strain variance.

#### 3.2.1 Genome‐wide significant QTL on chromosome 4 associated with preference for an auditory‐tactile‐visual reward

##### *Oss1* behavioral QTL

**Figure 2:**
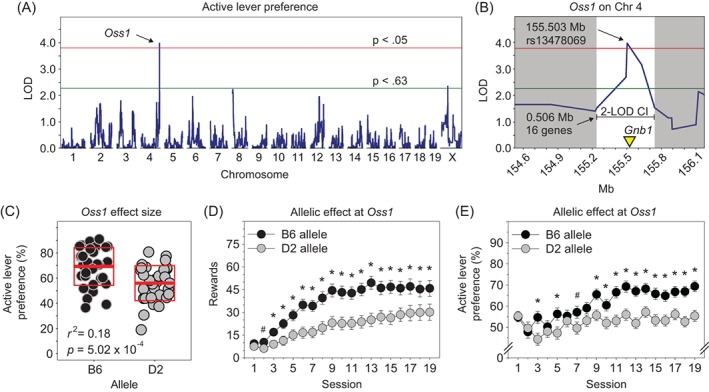
*Oss1* behavioral QTL associated with preference for an auditory‐tactile‐visual stimulus in an operant sensation seeking assay. A, Genome‐wide significant behavioral QTL on chromosome 4 (*Oss1*) associated with active lever preference on the final testing session of an operant sensation seeking assay. B, The 2‐LOD confidence interval for *Oss1* was 506 kilobases and contained 16 protein coding genes. C, *Oss1* accounted for 18% of the variance on active lever preference during the final session. D, E, Across sessions, mice with the B6 allele at *Oss1* received significantly more rewards and exhibited significantly greater active lever preference relative to mice with the D2 allele. *P < 0.05; #*P* < 0.10

Using GeneNetwork, we performed whole genome interval mapping using active lever preference on the final OSS session (ie, session 19). We identified a significant QTL (Figure 2A,B) on chromosome 4 with a peak locus of 155.503 Mb (LOD = 3.96, *P* = 3.20 × 10^−2^). The 2‐LOD CI was 155.236 to 155.742 Mb which encompassed 16 protein coding genes. This QTL accounted for 18% of the variance on active lever preference (Figure 2C). The marker at the QTL peak was rs13478069 which is located within *Gnb1*. Henceforth, we refer to this QTL as OSS QTL 1 (*Oss1*). Notably, we identified *Oss1* when using multiple imputation (above) and when dropping all mice that did not respond on the final session (LOD = 4.36, *P* = 1.00 × 10^−2^). The peak marker and CI were identical for the two methods.

Because active lever preference had stabilized by session 11, we performed a principal component analysis in GeneNetwork using active lever preference on each of the final nine sessions (sessions 11‐19) to determine if variance common to these sessions drove *Oss1* or if variance unique to session 19 was required to identify *Oss1*. This analysis produced a single principal component that was strongly correlated with active lever preference on sessions 11 to 19 (mean of nine Pearson correlation coefficients: *M* = 0.83, SD = 0.05). When we mapped this principal component, we again identified *Oss1*, this time with an even stronger LOD score (LOD = 4.69, *P* = 1.20 × 10^−2^). The peak marker was identical to those listed above for the multiple imputation and pairwise deletion methods used on session 19. Rank orders of strains on sessions 11 to 19 were strongly and positively intercorrelated (mean of 36 Spearman correlation coefficients: *M* = 0.65, SD = 0.09). When considering only the final five sessions, the intercorrelations were similar (mean of 10 Spearman correlation coefficients: *M* = 0.71, SD = 0.06). Collectively, these findings indicate that the variance in active lever preference that was common across sessions following stabilization of active lever preference on session 11 drove the *Oss1* QTL on chromosome 4.

##### Genetic dissociation of preference for an auditory‐tactile‐visual reward in the OSS paradigm from food‐rewarded pairwise discrimination learning

To assess the possibility that active lever preference in the OSS assay, once stabilized, reflects general operant learning rate or operant discrimination ability rather than preference for sensory stimuli, we calculated genetic correlations of active lever preference in the OSS paradigm on the final session with the following three variables which are publicly available on GeneNetwork: (a) Sessions to discriminate between rewarded and unrewarded stimuli in touchscreen visual discrimination learning; (b) Number of errors during touchscreen visual discrimination learning (c) Trials to criterion during acquisition of an operant nose‐poke spatial discrimination (GeneNetwork record IDs: 16204, 16208 and 12730).^29^, ^30^ Active lever preference in the OSS assay was not genetically correlated with any of these measures of food‐rewarded operant discrimination learning: (a) *r* = −0.02, *P* = 0.93, n = 22; (b) *r* = 0.00, *P* = 0.98, n = 22 and (c) *r* = −0.09, *P* = 0.59, n = 42.

##### Effects of the B6 and D2 alleles at *Oss1* on OSS

To assess the effect of genotype at *Oss1*, we grouped mice from the 62 BXD strains and two founder strains by the B6 (n = 124) or D2 (n = 123) allele at the peak of *Oss1* (B6‐ and D2‐allele groups, respectively). Using the multiple imputation data set, we performed a repeated measures anova using active lever preference as the dependent measure. Session (1‐19) was a within‐subjects factor. Sex and genotype at *Oss1* were between‐subjects factors. To assess the effect of *Oss1* on number of rewards, we performed an identical anova using rewards as the dependent measure.

Mice with the B6 allele at *Oss1* exhibited significantly greater active lever preference relative to mice with the D2 allele at *Oss1* as indicated by a main effect of allele (*F* (1, 243) = 14.84, *P* = 1.50 × 10^−4^, *η*_p_^2^ = 0.05). This effect varied as a function of session as indicated by a significant allele × session interaction (Figure 2E) (*F* (11.39, 2767.71) = 2.77, *P* = 1.17 × 10^−3^, *η*_p_^2^ = 0.01). Post hoc tests indicated that active lever preference of the B6‐allele group consistently improved across the 19 session OSS assay, whereas active lever preference of the D2‐allele group did not (Figure 2E). Consequently, active lever preference of the B6‐allele group was significantly greater than that of the D2‐allele group on all sessions during the final half of the assay. In addition to increased active lever preference, the B6‐allele group received significantly more rewards than the D2‐allele group on all but the first two sessions (Figure 2D) (allele × session: *F* (3.91, 949.91) = 3.50, *P* = 8.04 × 10^−3^, *η*_p_^2^ = 0.01). There was no interaction of sex and *Oss1* allele on active lever preference or number of rewards.

##### *Gnb1* is a positional candidate for *Oss1*

**Figure 3:**
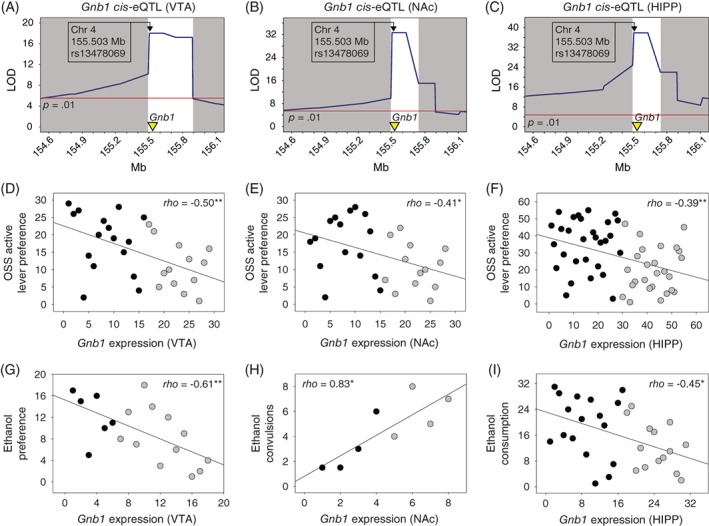
Expression of *Gnb1* in reward‐related brain regions is associated with preference for an auditory‐tactile‐visual stimulus and multiple ethanol phenotypes. A‐C, *Gnb1* is a positional candidate for *Oss1*. Genome‐wide significant *cis‐*eQTL associated with expression of *Gnb1,* which encodes the G protein β1 subunit, were identified in reward‐related brain regions including the ventral tegmental area, nucleus accumbens, hippocampus and prefrontal cortex (prefrontal cortex not shown). The peak marker for these *cis‐*eQTL is located within *Gnb1* and is identical to the peak marker for the *Oss1* behavioral QTL (Figure 2). D‐I, Expression of *Gnb1* in these brain regions is genetically correlated with preference for an auditory‐tactile‐visual stimulus and multiple ethanol phenotypes. Data points represent BXD strain means. Black and gray colors signify the B6 and D2 alleles, respectively, at the peak of the *Oss1* QTL. **P < 0.01; *P < 0.05

Positional candidates for *Oss1* were *Gnb1*, *Ssu72*, *Faap20* and *Prkcz*. The strongest candidate for *Oss1* was *Gnb1. Gnb1* exhibited significant *cis*‐eQTL in the VTA, NAc, HIPP (Figure 3A‐C) and PFC (data not shown). Notably, the peak marker for the *Oss1* behavioral QTL (Figure 2B), which is located within *Gnb1*, was identical to the peak marker for all *Gnb1 cis*‐eQTL.

*Gnb1*, which encodes subunit β1 of the guanine nucleotide binding protein (G protein), has been proposed as a positional candidate influencing, and influenced by, the use of alcohol and other addictive substances.^31^, ^32^, ^33^, ^34^, ^35^ Collectively, these findings and those from the present study suggest a pleiotropic effect of *Gnb1* on sensation seeking and substance use. To test this hypothesis, we examined the genetic correlations among *Gnb1* expression in reward‐related brain regions, OSS active lever preference and behaviors related to substance use. *Gnb1* expression in VTA, NAc and HIPP significantly covaried with active lever preference (Figure 3D‐F) and alcohol‐related phenotypes (Figure 3G‐I): ethanol preference in the two bottle choice paradigm,^36^ handling induced convulsions following ethanol injection,^37^ and ethanol consumption using the drinking in the dark paradigm (GeneNetwork record IDs: 10140, 11380 and 18877).

Previous studies indicate that the heterotrimeric G protein coupled metabotropic glutamate receptor 5 (mGluR5) influences OSS in mice^17^, ^38^ and novelty seeking in humans.^39^ Metabotropic glutamate receptors, including mGluR5 and mGluR7, have also been shown to affect alcohol and other drug use.^17^, ^40^, ^41^, ^42^, ^43^ As *Gnb1* encodes the β1 subunit of heterotrimeric G proteins, one hypothesis is that *Gnb1* influences OSS and alcohol use through a mechanism involving mGluR5 or other metabotropic glutamate receptors. To assess the genetic relationship between these variables, we examined genetic correlations of *Grm1 to Grm8* (which encode mGluR1‐mGluR8) expression in reward‐related brain regions and (a) *Gnb1* expression, (b) OSS active lever preference and (c) alcohol use. In the HIPP, expression of *Grm5* was significantly genetically correlated with OSS active lever preference on the final testing session (*rho* = 0.39; *P* = 0.002; n = 55), *Gnb1* expression (*rho* = −0.29; *P* = 0.01; n = 71) and ethanol consumption using the drinking in the dark paradigm (*rho* = 0.39; *P* = 0.02; n = 33) (GeneNetwork record ID: 20335). Expression of *Grm7* was significantly genetically correlated with active lever preference on the final session in the VTA (*rho* = 0.43; *P* = 0.02; n = 29), NAc (*rho* = −0.54; *P* = 0.002; n = 28), HIPP (*rho* = 0.31; *P* = 0.02; n = 55) and PFC (*rho* = 0.43; *P* = 0.04; n = 22). *Grm7* expression was significantly genetically correlated with *Gnb1* expression in the VTA (*rho* = −0.44; *P* = 0.006; n = 37).

#### 3.2.2 Genome‐wide significant QTL on chromosome 13 associated with lever pressing but not preference for an auditory‐tactile‐visual reward

##### *Oss2* behavioral QTL

**Figure 4:**
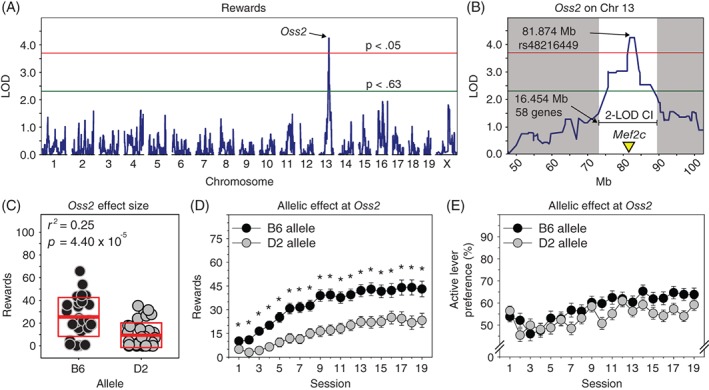
*Oss2* behavioral QTL associated with number of rewards but not active lever preference in an operant sensation seeking assay. A, Genome‐wide significant behavioral QTL on chromosome 13 (*Oss2*) associated with number of rewards, active levers presses or inactive lever presses on multiple sessions (1, 2, 3, 4, 5 and 9) of an operant sensation seeking assay when mice were still learning the response‐reward contingencies. Data from rewards on session 5 is shown. B, The 2‐LOD confidence interval for *Oss2* spanned 16.5 Mb of chromosome 13 and contained 58 protein coding genes. C, *Oss2* accounted for 25% of the variance on number of rewards. D, E, As seen with *Oss1*, mice with the B6 allele at *Oss2* received significantly more rewards relative to mice with the D2 allele. In contrast to *Oss1*, the allele at *Oss2* did not significantly affect active lever preference. *P < 0.05

Across multiple sessions and multiple variables, we identified a significant QTL (Figure 4) located on chromosome 13 with a peak at 81.874 Mb. This same genome‐wide significant QTL was identified for rewards (sessions 1, 2 and 5), active lever presses (sessions 2, 3, 4, 5 and 9) and inactive lever presses (sessions 2 and 5). Here, we describe this QTL using number of rewards on session 5 as the dependent measure.

We performed whole genome interval mapping using rewards on session 5 as the dependent measure. Four BXD strains (BXD6/TyJ, BXD67/RwwJ, BXD68/RwwJ and BXD101/RwwJ) were identified by GeneNetwork as outliers (z‐scores ≥ |2.5|) and were automatically dropped from the QTL analysis and excluded from subsequent analyses. We identified a significant QTL (Figure 4A,B) on chromosome 13 with a peak locus of 81.874 (LOD = 4.25, *P* = 2.40 × 10^−2^). This QTL accounted for 25% of the variance on number of rewards (Figure 4C). The 2‐LOD CI was 72.969 to 89.423 Mb and encompassed 58 protein coding genes. The marker at the QTL peak was rs48216449. This marker was genome‐wide significant when mapping rewards (sessions 1, 2 and 5), active lever presses (sessions 2, 3, 4, 5 and 9) and inactive lever presses (sessions 2 and 5). Henceforth, we refer to this QTL as OSS QTL 2 (*Oss2*).

##### Effects of the B6 and D2 alleles at *Oss2* on OSS

To assess the effect of genotype at *Oss2*, we grouped mice from the 58 BXD strains and two founder strains by the B6 (n = 104) or D2 (n = 127) allele at the peak of *Oss2* (B6‐ and D2‐allele groups, respectively). We performed a repeated measures anova using number of rewards as the dependent measure. Session (1‐19) was a within‐subjects factor. Sex and genotype at *Oss2* were between‐subjects factors. To assess the effect of *Oss2* on preference for the active lever, we performed an identical anova using active lever preference as the dependent measure.

Mice with the B6 allele at *Oss2* received significantly more rewards relative to mice with the D2 allele at *Oss2* (Figure 4D) as indicated by a main effect of allele (*F* (1, 227) = 20.29, *P* = 1.10 × 10^−5^, *η*_p_^2^ = 0.08). The allele × session interaction was marginally significant (*F* (3.53, 801.74) = 2.47 *P* = 5.07 × 10^−2^, *η*_p_^2^ = 0.01). Sex did not influence the effect of *Oss2* allele on number of rewards. There was no significant main effect of *Oss2* genotype or interaction involving *Oss2* genotype on active lever preference (Figure 4E).

##### *Mef2c* is a positional candidate for *Oss2*

**Figure 5:**
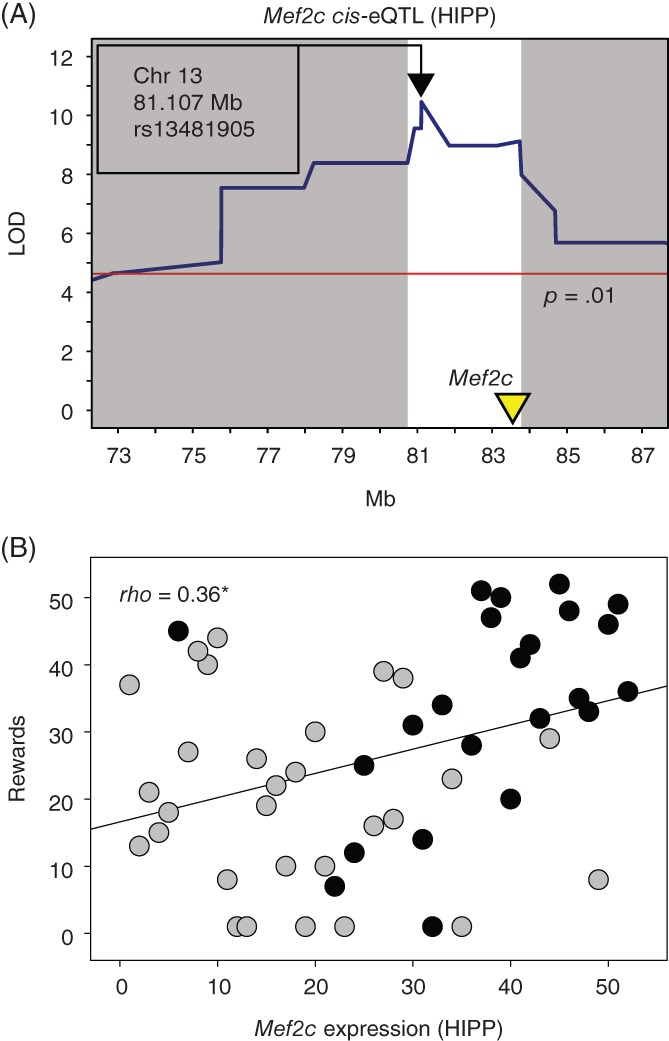
Expression of *Mef2c* is associated with number of rewards in an operant sensation seeking assay. *Mef2c* is a positional candidate for *Oss2*. A, Genome‐wide significant *cis‐e*QTL associated with expression of *Mef2c,* which encodes myocyte enhancer factor 2C*,* was identified in the hippocampus. B, Expression of *Mef2c* mRNA in the hippocampus was positively genetically correlated with number of rewards on an operant sensation seeking assay. Data points represent BXD strain means. Black and gray colors signify the B6 and D2 alleles, respectively, at the peak of the *Oss2* QTL. *P < 0.05

Positional candidates for *Oss2* were *Mef2c, Glrx1, Arsk, 2210408I21Rik, Kiaa1024l, Rhobtb3 and Adgrv1*. The strongest candidate was *Mef2c* (myocyte enhancer factor 2C) which is involved in transcriptional processes controlling synapse number^44^, ^45^ and cognitive function.^46^ *Mef2c* exhibited significant *cis*‐eQTL in the HIPP, and expression of *Mef2c* mRNA in this region covaried significantly with reward number (Figure 5). Previous studies of *Mef2c* informed the nomination of this gene to positional candidate status. Specifically, embryonic deletion^45^ and postnatal deletion of *Mef2c* in the forebrain, including the HIPP,^44^ resulted in deficits in motor coordination and locomotor hyperactivity. These deficits may have resulted in reduced species typical exploration and impaired lever pressing ability in the present study without effects on reward processing.

## 4 DISCUSSION

In the present study, we used a systems genetics approach to identify novel gene candidates driving behavior in the OSS assay. We quantified OSS in 247 mice (120 males, 127 females) from 62 BXD strains and the C57BL/6J and DBA/2J founder strains (Figure 1). Using these data, we identified genomic regions on chromosomes 4 (*Oss1*) and 13 (*Oss2*) associated with distinct behavioral components of OSS (Figures 2 and 4). Using publicly available behavioral data and mRNA expression data from brain regions involved in reward processing, we identified (a) genes within behavioral QTL exhibiting genome‐wide significant *cis*‐eQTL and (b) genetic correlations among mRNA expression, OSS phenotypes and substance use phenotypes (Figures 3 and 5). From these analyses, we nominated positional candidates (*Gnb1* and *Mef2c*) for behavioral QTL associated with distinct OSS phenotypes. Genetic covariation of *Gnb1* expression, preference for an auditory‐tactile‐visual stimulus and multiple ethanol phenotypes suggest a pleiotropic effect of *Gnb1* on sensation seeking and ethanol use (Figure 3).

### 4.1 Genome‐wide significant QTL on chromosomes 4 and 13 are associated with distinct behavioral components of OSS

We identified a behavioral QTL on chromosome 4 (*Oss1*) associated with active lever preference on the final OSS testing session after the task was well learned. We identified a second QTL on chromosome 13 (*Oss2*) associated with number of rewards, active lever presses and inactive lever presses during early sessions when mice were still learning the response‐reward contingencies. Importantly, the effects of the allele (B6 or D2) at the peak of these QTL were behaviorally distinct. Specifically, the B6 allele at the peak of *Oss1* potentiated both reward number and active lever preference. This pattern of responding suggests that the allele driving *Oss1* affects reward processing associated with the presentation of the compound auditory‐tactile‐visual stimulus. In contrast to *Oss1*, the B6 allele at the peak of *Oss2* potentiated reward number but did not significantly influence active lever preference. This pattern of responding suggests that the allele driving *Oss2* affects the probability of pressing a lever (both active and inactive), possibly through increased hyperactivity or reduced motor coordination, but not reward processing associated with the presentation of the auditory‐tactile‐visual stimulus.

Notably, we observed wide between‐strain variance on active lever preference (Figure 2C) which likely reflects the full spectrum of affective responses to sensory stimuli presentation including reinforcement, absence of reinforcement and aversion. In this regard, the very low active lever preference scores in some BXD strains may be explained by sensory stimuli aversion. Specifically, mice experiencing aversion to the compound auditory‐tactile‐visual stimulus would avoid the area around the active but not inactive lever. Locomotion in the chamber would therefore result in a higher percentage of incidental inactive lever presses resulting in a below‐chance active lever preference score.

In the present study, we used all tested mice for QTL mapping, irrespective of whether they met commonly used acquisition criteria (eg, 70% of responses on the active lever for three sessions). By doing this, we leveraged the full range of phenotypic variation which maximized power for QTL mapping. In a separate analysis, we excluded all mice that did not meet these acquisition criteria (n = 136). We did not identify genome‐wide significant QTL using this data set. This was likely due to the reduced statistical power from the combination of reduced sample size and reduced between‐strain variance.

Habituation of reinforcer effectiveness and variation in the rate at which this occurs across types of reinforcers may be an important factor underlying addiction.^10^, ^11^ In the OSS assay, robust within‐session habituation to the reinforcing effects of purely visual stimuli has been observed.^10^, ^11^ In the present study, we did not observe within‐session habituation to the reinforcing effects of the auditory‐tactile‐visual stimulus; this may have been due to the relatively high intensity of this compound, multimodal stimulus. Stimulus intensity is an important consideration because very intense stimuli may yield no observable response decrement.^47^ Moreover, flash frequency of visual stimuli and the duration of their presentation were randomized within‐session which likely further reduced habituation. We did not design the operant protocol such that we could use flash frequency or duration of stimulus presentation as independent variables and were unable to test hypotheses associated with these variables. In addition to reinforcer habituation, flash frequency may be relevant to the biology of autism due to stimulus hypersensitivity in this disorder.^48^ In future studies, use of stimulus modality and duration of stimulus presentation as independent variables may enable a more nuanced understanding of the genetic factors driving habituation of reinforcer effectiveness and how these overlap with the genetic factors driving sensation seeking.

### 4.2 *Gnb1* is a positional candidate for preference for an auditory‐tactile‐visual reward in the OSS behavioral assay

Systems genetics analysis suggests that *Gnb1* is the strongest gene candidate for the *Oss1* QTL which is associated with preference for an auditory‐tactile‐visual stimulus. Specifically, we identified genome‐wide significant *Gnb1 cis*‐eQTL in brain regions associated with reward processing (VTA, NAc, PFC and HIPP). Notably, the peak marker for the *Oss1* behavioral QTL (Figure 2B), which is located within *Gnb1*, was identical to the peak marker for all *Gnb1 cis*‐eQTL (Figure 3A‐C). Moreover, expression of *Gnb1* mRNA was significantly negatively correlated with active lever preference in the VTA, NAc and HIPP (Figure 3D‐F). Analysis of allelic effects (Figure 2C‐E) at the peak of *Oss1* indicates that the B6 allele potentiates active lever preference relative to the D2 allele. These data suggest that a SNP within or around *Gnb1* differentially controls expression of *Gnb1*, and the consequent attenuated expression in BXD strains with the B6 allele potentiates responding on the active but not inactive lever. This results in potentiation of both active lever preference and number of delivered rewards in these mice.

Regarding the mechanism underlying this effect, *Gnb1* encodes the β1 subunit of heterotrimeric G proteins which are composed of an α, β and γ subunit.^49^ Multiple subtypes of each subunit are known including at least five β subunits (β1‐β5), each encoded by a distinct gene (*Gnb1*‐*Gnb5*).^50^ G protein mediated signaling cascades underlie a broad range of cellular processes including the inhibitory actions of many neurotransmitters.^51^, ^52^ Combined with findings from the present study, these data suggest that heritable variation in *Gnb1* expression affects the drive to experience sensory stimuli by affecting G protein mediated intracellular signaling pathways, possibly in concert with metabotropic glutamate receptors and through inhibition of neurotransmission in brain regions involved in reward processing.

### 4.3 *Mef2c* is a positional candidate for reward number but not preference for an auditory‐tactile‐visual reward in the OSS behavioral assay

Systems genetics analysis suggests that *Mef2c* is the strongest gene candidate for the *Oss2* QTL which is associated with number of rewards and lever presses but not preference for the active lever in the OSS assay. Specifically, we identified genome‐wide significant *Mef2c cis*‐eQTL in the HIPP and a significant positive correlation of *Mef2c* mRNA expression and rewards in the same region (Figure 5). Analysis of allelic effects at the peak of *Oss2* (Figure 4C‐E) indicates that the B6 allele potentiates reward number, but not preference for the reward, relative to the D2 allele. These findings suggest that a SNP near *Mef2c* differentially controls expression of *Mef2c*, and the consequent increased expression in BXD strains with the B6 allele potentiates lever pressing equally on both the active and inactive lever.

Regarding the mechanism underlying this effect, embryonic deletion^45^ and postnatal deletion of *Mef2c* in the forebrain, including the HIPP,^44^ result in motor coordination deficits and locomotor hyperactivity. Therefore, in the present study, it is possible that (a) increased hyperactivity resulted in reduced species typical exploration which caused an overall reduction in lever pressing or (b) motor coordination deficits impaired lever pressing ability or changed lever pressing dynamics which directly caused an overall reduction in lever pressing. Either of these behavioral changes alone could account for observed differences in reward number without invoking effects on reward processing of auditory‐tactile‐visual stimuli. However, it remains possible that increased reward number alone, without an increase in active lever preference, could indicate an effect of *Mef2c* on reward processing of sensory stimuli. Moreover, it is possible that a significant effect of *Mef2c* on active lever preference could emerge with (a) additional OSS fixed‐ratio 1 testing beyond 19 days, (b) additional testing using a distinct OSS protocol (eg, progressive ratio), (c) use of more strains or a larger within‐strain sample size to increase statistical power or (d) use of a more genetically diverse mouse resource such as the Collaborative Cross RI panel or Diversity Outbred population.^6^, ^53^

### 4.4 Hereditary and environmentally‐induced variation in *Gnb1* expression in reward circuitry may partially underlie the observed relationship between sensation seeking and substance abuse

To our knowledge, the present study provides the first evidence that *Gnb1* affects the drive to experience sensory stimulation. However, several studies suggest that heritable variation in G proteins in general,^54^ and the product of *Gnb1* in particular,^34^ influence substance use. Moreover, several studies indicate that *Gnb1* expression is itself influenced by environmental perturbations including exposure to addictive substances.^31^, ^32^, ^33^, ^35^ Together with findings from the present study, these data suggest that heritable and environmentally‐induced variation in *Gnb1* expression are key drivers of the genetic and phenotypic covariation of substance use and sensation seeking which has been observed in human and preclinical studies. We tested this hypothesis by examining genetic correlations among *Gnb1* expression in brain regions involved in reward, active lever preference and behavioral phenotypes involving addictive substances. We found significant genetic correlations among these phenotypes. Specifically, *Gnb1* mRNA expression in the VTA, NAc and HIPP was genetically correlated with OSS active lever preference (Figure 3D‐F) and ethanol phenotypes from multiple studies and labs (Figure 3G‐I). These data support the hypothesis that heritable variation in *Gnb1* partially underlies both sensation seeking and ethanol use. Reverse genetics validation studies will be needed to evaluate the full range of effects of *Gnb1* perturbation on ethanol and sensation seeking phenotypes (eg, dose‐response curves, extinction, reinstatement, escalation of responding over time and within‐session habituation of reinforcer effectiveness) and to determine if similar effects on psychostimulants and opiates can be observed.
